# Supplementary material for: On the connection between real-world circumstances and online player behaviour: The case of EVE Online
Source: PLoS One. 2020 Oct 21;15(10):e0240196. doi: 10.1371/journal.pone.0240196 (PMC7577455; doi:10.1371/journal.pone.0240196)
Supplement: Attachment — Submitted filename: ResponseToReviewers.pdf [file pone.0240196.s002.pdf]

REBUTTAL LETTER

Reviewer 1: Review report for "On the connection between real-world circumstances and online player behaviour: the case of EVE Online" by Belaza et al.

This paper studies the connection between real-world circumstances and behaviors of online players by a case study of the EVE Online game. Specifically, the authors explored the influence of the real-world context in which players live on their in-game behavior. They found a negative relation between in-game aggressiveness and real-world aggressiveness in the players country of residence, showing that players experiencing violence in daily life have a tendency to be less aggressive in online games. Moreover, players from more peaceful countries tend to maintain more friends relatively to those living in more violent countries. The authors also found some correlations between in-game trading and real-world variables reflecting the macroeconomic context where players live, for example, there are correlations between unemployment rates and money-conscious in-game trading behavior.

Understanding the connections between real-world circumstances and online behaviors is an interesting and important issue, which has implications for better understanding human behaviors and designing better experiments with a variety of settings. I think this paper considers an interesting research question, the results and analyses are convincing, and the structure of the paper is good. In my view, this paper deserves a final publication. In the following, I would like to provide some suggestions and comments, which may be helpful for the authors to further strength their paper.

OUR RESPONSE: Thank you for finding our paper interesting and for recommending a final publication. We appreciate the careful reading of the manuscript and the highly valuable feedback. We have addressed all recommendations and suggestions for further improvement in preparing the revised manuscript and are confident that the amendments further improved the overall quality of the manuscript.

R1.1 In the abstraction section, the authors presented that "Virtual worlds involving social and economic interactions are particularly useful to test social and economic theories". I think this is particularly interesting considering that analyzing behaviors in virtual worlds has relatively low cost and virtual worlds are more flexible in implementing experiments to test social and economic theories if they are well representations of offline worlds. The authors also mentioned in the first paragraph of the introduction section that "A major question, however, is whether the data extracted from virtual worlds are representative enough to study real-world socioeconomic phenomena and whether they capture real-world human behaviour". There are some previous works that can better support the authors' narratives in related content, for example, using online posts and mobile communications to infer individual economic status [Luo, S., Morone, F., Sarraute, C., Travizano, M., Makse, H. A. (2017). Inferring personal economic status from social network location. Nature communications, 8(1), 1-7], and infering socioeconomic status from large-scale online data sources [Gao, J., Zhang, Y. C., Zhou, T. (2019). Computational socioeconomics. Physics Reports, 817, 1-104]. The authors are suggested to cover a relative broad branch of literature and present a more general introduction of the background of this paper.

OUR RESPONSE: In order to cover a broader branch of literature, we have added the two mentioned references and an extra paragraph in the Introduction. Those discuss the scientific endeavour to establish reliable connections between digital data and real-world socioeconomic phenomena.

R1.2 On page 2, the authors presented that "Using EVE data, Carter has conducted a

study of the use of propaganda during war times as an essential factor for maintaining the morale [28]". It would be better to keep the typical citing format as the authors used. That is "Using EVE data, Carter [28] has conducted a study xx". On page 3, the authors presented that "To play EVE Online, a player needs to pay a subscription fee of 15 USD or 15 EUR per month, or spend the equivalent amount by buying an item (called PLEX) inside the game". I am wondering how the subscription fee will affect the enrollments of the EVE Online. For some low-income countries, the sampling rate of all population may be different. It would be necessary to present a new figure showing the number of users for each country, and the share to all population in each country. I am wondering if the sampling bias will affect the observations.

OUR RESPONSE: Thanks for calling our attention to the inconsistent use of the citing format - we have implemented the suggested change. We have created a figure (included in this document) with the number of accounts per capita for countries with more than 100 accounts in EVE Online. We have added a discussion to the Subsection EVE Online that mentions that per capita there tends to be more players from high-income than from low-income countries. We have also included some information about the number of players relative to the total population.

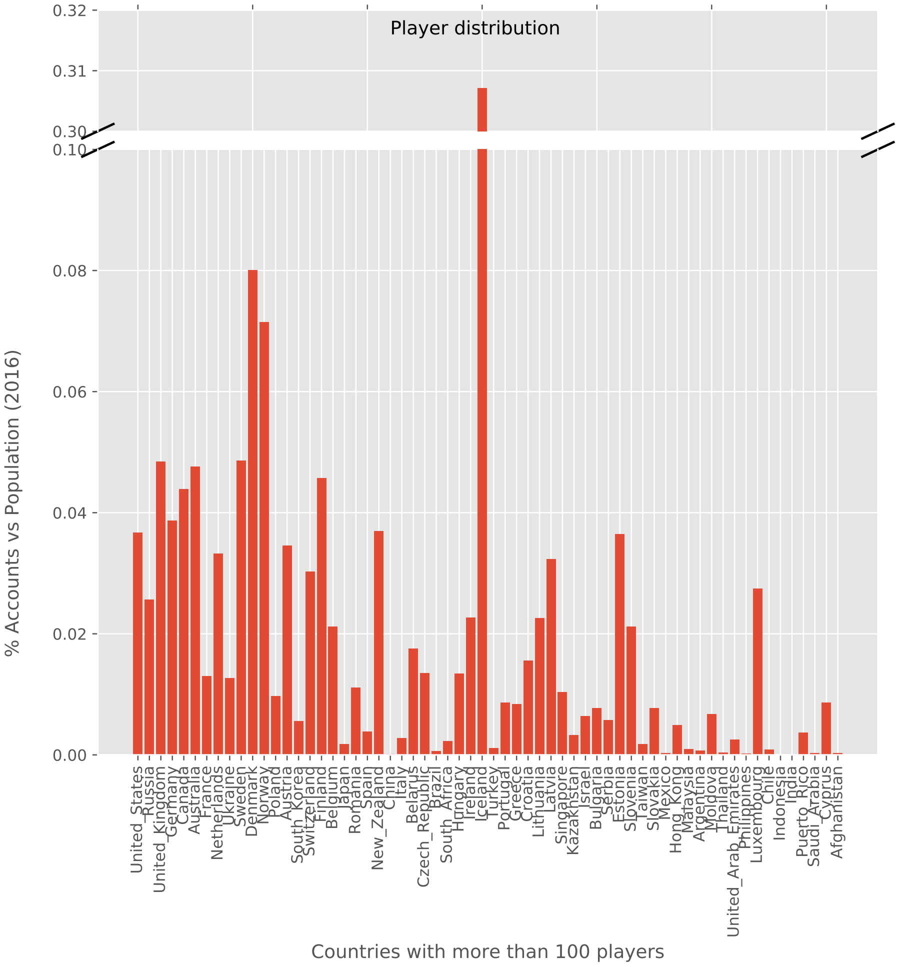

R1.3 On page 3, the authors presented that “The analysis presented in this work is based on collected EVE Online data from December 2011 to December 2016 (61 months) for the trade activity and the year 2016 for the social and economic activity”. It seems that the authors used cumulative data across their study, however, I am wondering if there are any temporal changes of these patterns observed in this paper, for example, the correlations between online game behavioral features and real-world economic indicators in the year 2014, and how the results evolve over time. Regarding the two variables: (i) Added As Negative” and (ii) Added As Positive, I think there are also temporal changes, for example, a player can be added as negative and as positive at different periods considering the online game is a dynamitic systems and friendships among players can change quickly. I am wondering how the authors capture these evolving characteristics.

OUR RESPONSE: Our focus is on the comparison across countries and in order to include a good sample of different countries we need to aggregate the data from individual players over time. A temporal analysis that focuses on the socioeconomic behavior of individuals would only be feasible for a few countries with a large amount of players and is beyond the scope of the present manuscript. In other words, the focus of our work is on connections between real-world and in-game socioeconomic behaviour at the collective country level. To this end we time aggregate over the data from all individual players from a particular country. For example, in the category of “aggressiveness” a player can be added a couple of times as “positive” and a few times as “negative” in the course of particular week. All these events add to the country profile. We have substantially extended the discussion of the data curation process in the Subsection *In-game country profile*. Thereby we have extended the description of the event space and how we do aggregate over time. Also at the end of the introduction we have inserted a discussion stressing that the focus of our study is on the collective country level. We aggregate over the in-game activities of all individual players from a certain country to infer a quantitative view of the average in-game socioeconomic behaviour at the country level.

R1.4 On page 4, the authors presented that “The latter three activities result in the generation of an in-game item that we transform into a numeric value using the market prices. Data are aggregated on a weekly basis, creating a specific player profile per week”. I think it remains unclear how the authors did the transformation into a numeric value. The authors are suggested to add more explanations to this point. On page 5, the authors presented that “Finally, the player’s profile is normalised by the sum of the eight adimensional measures of the in-game activities”. I am wondering if the method of normalization may affect the results and if the sum of the eight adimensional measures is treating measures with equal weights.

OUR RESPONSE: As suggested we have added more explanations about the data cleaning procedure at the end of the “In-game country profile” section. In a step-wise fashion we provide details about the data cleaning process that allowed us to create quantitative country profiles from the recorded players’ in-game activities.

R1.5 In Table 3, the authors presented country profiles for selected countries. I noticed that most of these countries are western countries. Maybe it is better to include some Eastern and Asian countries as well considering a diverse set of cultural backgrounds. In Fig 1, the authors presented the in-game similarity between countries. First, it would be better to show all country names in the x-axis as well.

Second, it would be better to reverse the color scheme, where red indicates positive value and blue indicates negative values.

OUR RESPONSE: (i) We have added the data for two non-Western countries to Table 3 (Japan and The Philippines). The figure has been updated in accordance with the reviewer's suggestions.

R1.6 In Table 5 and Table 6, it would be better to list Intercept blow other control variables but above the R2. Listing the variables of most interests in the top of the tables is a tradition. Also, No. observations should be listed above R2. In addition, the authors presented some convincing correlations among online behaviors and offline socioeconomic status. Yet, I am wondering if it is possible to identify some causal relationships using instrumental variables. It would be not easy due to the observational data, but I am wondering the possibility. It would be nice to add some related discussions in the discussion section.

OUR RESPONSE: Thanks for the suggestion: we have changed the ordering of the lines in Tables 5 and 6. We fully agree that establishing causal relationships using the instrumental variables is challenging. The possibility to study causal relationships is mentioned in the conclusion.

R1.7 The authors found that "players from safer countries behave on average more aggressively towards other players in the virtual world than players from unsafer countries". I am wondering if there are social and psychological explanations for this observation. In the reference section, it would be better to presented the full journal name for PNAS as "Proceedings of the National Academy of Sciences, U.S.A.". For other references, they are in very good shape of citing format.

OUR RESPONSE: Thanks for the suggestions. (i) We have inserted an extra paragraph in the Discussion (lines 390-397) and an extra reference [41]) from the psychology literature addressing the connection between violence in the real world and in video games. The conclusions of the study of [41] are in line with ours. We have added to the Discussion section some possible explanations quoted in [41]. Upon discussing the results of Table 5 in the Section "In-game player behaviour and the real-world socioeconomic environment" we have added possible social and psychological explanations. We have reworded parts of the Abstract so as to pay better attention to social and psychological explanations to the findings of our analyses.
(ii) We have replaced "PNAS" by "Proceedings of the National Academy of Sciences (USA)".

Reviewer 2

In this manuscript, authors analyze the influence of the real-world context in which players live on their in-game behavior. This work is of great interests, and the analysis is solid. I would recommend publication after the authors make proper minor modifications.

OUR RESPONSE: Thank you for finding our paper interesting and solid and for recommending publication. We have addressed the highly valuable recommendations of the report and are confident that this further improved the quality of the manuscript.

R2.1 It is better to highlight the contributions of this work and the difference between this work and the previous studies in the introduction.

OUR RESPONSE: We have added a number of references and paragraphs in order to cover a broader branch of literature and to present a more general introduction of the background of this paper. In the Introduction we have included a number of examples of studies that establish connections between digital data and

real-world socioeconomic phenomena. In the Discussion section we have inserted a paragraph discussing specific results from Psychology literature that looked into the connection between violence in games and in the real world. We also refer to the responses to R1.1 and R1.7.

R2.2 About the in-game country profile, data are aggregated on a weekly basis, creating a specific player profile per week. And the variables on social interaction, aggressiveness and production activity are calculated by the absolute quantity. I think the times players in different countries spend on online game may varies. Therefore, it is better to use the number of activities per unit of online time to calculated the variables, right?

OUR RESPONSE: This is an interesting suggestion. Unfortunately, the game developers could not provide us with information about the connection times for the individual players. The adopted data cleaning process, however, guarantees that activities from highly active players that stay online for long times, have a very heavy weight in the extracted country profiles.

R2.3 "For the country profile, the average of all the profiles of the players belonging to that country is taken and normalized by the standard score of all countries with at least 15 players in EVE online. ". Please provide more statistic analysis to support the threshold (15 players).

OUR RESPONSE: The threshold of a minimum of 15 players is a trade-off between data cleanliness and country coverage. We wish to have as many countries as possible in our analysis. The number of countries can be increased by lowering the threshold. For countries with few players, however, we obtain highly noisy signals. We have checked that increasing the threshold from 15 to a higher number does not markedly modify the results. The signal for countries with less than 15 players, however, tends to be very noisy. In order to further clarify this aspect of our analysis we have added explanations when we first mention the threshold of 15 players.

Journal Requirements:

When submitting your revision, we need you to address these additional requirements.

J1 Please ensure that your manuscript meets PLOS ONE's style requirements, including those for file naming.

OUR RESPONSE: CHECKED THIS

J2 Please include a brief description in your methods section of how you accessed/collected the data, and how your full dataset can be accessed by future researchers.

OUR RESPONSE: At the end of the "In-game country profile" we have added a step wise description of the data cleaning process. The data of the country profiles are made available for downloads from the authors' website.

J3 Please remove your figures from within your manuscript file, leaving only the individual TIFF/EPS image files, uploaded separately. These will be automatically included in the reviewers' PDF.

OUR RESPONSE: DONE

J4 Thank you for stating the following financial disclosure:

'The funders had no role in study design, data collection and analysis, decision to publish, or preparation of the manuscript.'

At this time, please address the following queries:

Please clarify the sources of funding (financial or material support) for your study. List the grants or organizations that supported your study, including funding received from your institution. State what role the funders took in the study. If the funders had no role in your study, please state: "The funders had no role in study design, data collection and analysis, decision to publish, or preparation of the manuscript." If any authors received a salary from any of your funders, please state which authors and which funders. If you did not receive any funding for this study, please state: "The authors received no specific funding for this work."

OUR RESPONSE: We state that "The funders had no role in study design, data collection and analysis, decision to publish, or preparation of the manuscript."
